# Treatment of peritoneal fibrosis: Therapeutic prospects of bioactive Agents from Astragalus membranaceus

**DOI:** 10.3389/fphar.2024.1347234

**Published:** 2024-05-15

**Authors:** Ying Huang, Chenling Chu, Yuanyuan Mai, Yue Zhao, Luxi Cao, Shuiyu Ji, Bin Zhu, Quanquan Shen

**Affiliations:** ^1^ School of Public Health, Hangzhou Medical College, Hangzhou, China; ^2^ Urology and Nephrology Center, Department of Nephrology, Zhejiang Provincial People’s Hospital, Affiliated People’s Hospital, Hangzhou Medical College, Hangzhou, China; ^3^ Department of Clinical Medicine and Stomatology, School of Hangzhou Normal University, Hangzhou, China; ^4^ Basic Medical Sciences, Hangzhou Medical College, Hangzhou, China; ^5^ Department of Nephrology, Zhejiang Provincial People’s Hospital Bijie Hospital, Bijie, China

**Keywords:** peritoneal fibrosis, Astragalus membranaceus, astragaloside, Astragalus saponin, Astragalus polysaccharide, inflammation peritoneal fibrosis

## Abstract

Peritoneal dialysis is one of the renal replacement treatments for patients with end-stage renal disease. Peritoneal dialysis-related peritoneal fibrosis is a pathological change in peritoneal tissue of peritoneal dialysis patients with progressive, non-suppurative inflammation accompanied by fibrous tissue hyperplasia, resulting in damage to the original structure and function, leading to peritoneal function failure. Currently, there is no specific drug in the clinic. Therefore, it is necessary to find a drug with good effects and few adverse reactions. Astragalus membranaceus (AMS) is the dried root of the *Astragalus membranaceus* (Fisch.) Bge. AMS and its active ingredients play a significant role in anti-inflammation, anti-fibrosis, regulation of immune function and regulation of blood pressure. Studies have shown that it can alleviate peritoneal fibrosis by reducing inflammatory response, inhibiting oxidative stress, degrading extracellular matrix deposition, regulating apoptosis, and regulating Transforming Growth Factor-β. The author summarized the relationship between AMS and its active ingredients by referring to relevant literature at home and abroad, in order to provide some theoretical basis for further clinical research.

## 1 Introduction

Peritoneal dialysis (PD) is an effective kidney replacement therapy for patients with end stage renal disease. Compared with hemodialysis, PD has advantages such as low cost, preservation of residual renal function, and stable hemodynamics (P. K.-T. Li et al., 2017). However, long-term PD treatment will lead to peritoneal structure and function disorders, leading to peritoneal fibrosis (PF) and peritoneal failure, resulting in the termination of PD (Zhou et al., 2016). PF has two interacting parts, including the fibrotic process itself and inflammatory progression, in other words, the occurrence process of PF is closely related to peritoneal inflammation (Hu et al., 2023), epithelial-mesenchymal transition (EMT) (Giri et al., 2023; Kunoki, 2023), oxidative stress (Zhao, Shi, et al., 2020), apoptosis (B. Wang et al., 2018), and micro RNA (Gohlke et al., 2022). At present, there are no specific drugs for PF in clinic, mainly glucocorticoids and/or immunosuppressants, whose purpose is to alleviate symptoms and delay the progression of fibrosis as much as possible (Gong et al., 2022; Zhu et al., 2022). Therefore, it is imperative to actively explore the mechanism of PD-induced PF, seek mature and effective targets for the prevention and treatment of PF, and develop new PF treatment methods.

According to the theory of traditional Chinese medicine, PF belongs to the category of “micro syndrome”, the formation of syndrome is mainly caused by Qi accumulation and blood stasis. The treatment should be based on the method of promoting Qi and promoting blood circulation, and the long-term disease consumption of Qi should be supplemented by the prescription of tonifying Qi. Modern pharmacology believes that AMS contains many active ingredients such as polysaccharides, saponins, flavonoids, organic acids and trace elements, and plays a significant role in anti-inflammation, anti-fibrosis, regulation of immune function and regulation of blood pressure (Lau et al., 2012; Kai et al., 2015). At the same time, AMS and its active ingredients have low toxicity and few adverse reactions, and play a positive role in the prevention and treatment of PD-related PF (Agyemang et al., 2013). Therefore, in order to clarify the potential application of AMS and its active components in the prevention and treatment of PF, this paper will review its anti-PD-related PF action and its mechanism, in order to provide scientific support for future PF treatment research direction. Date information was mainly collected from PubMed, Web of Science and CNKI databases (up to April 2024). The anti-peritoneal fibrosis effects of *Astragalus membranaceus* (Fisch.) Bge and its active components were reviewed in this paper. Search keywords for this type of work include: " Astragalus membranaceus ", “Astragaloside”, “Astragalus saponin”, “total Astragalus saponin”, “Astragalus polysaccharide” and them corresponding subject terms, and “peritoneal fibrosis” or “retroperitoneal fibrosis".

## 2 Mechanism of peritoneal fibrosis

The traditional peritoneal dialysate used lactate as buffer, supplemented with different concentrations of glucose, low pH, high glucose and hyperosmolar are its characteristics (Nakano et al., 2020). The solution will produce a large number of glucose degradation products (GDPs) and advanced glycation end products (AGEs) under high temperature. It leads to inflammation and oxidative stress (Khalid et al., 2022), so the inflammatory reaction runs through PF. On the one hand, peritoneal contact with peritoneal permeate with poor biocompatibility causes micro-inflammation in the peritoneum (Alqaedi et al., 2021); on the other hand, inflammatory injury also stimulates peritoneal mesodermal cells to release inflammatory factors, such as tumor necrosis factor-α (TNF-α), interleukins (ILs), interferons, etc., cause inflammatory response and induce inflammatory cells (such as monocytes, macrophages, neutrophils, etc.) to gather at the damaged site, further releasing inflammatory mediators and reactive oxygen species (ROS),etc., leading to increased intraperitoneal inflammatory response (Henderson et al., 2020). At the same time, the excessive release of inflammatory factors will also cause changes in the extracellular matrix (ECM), leading to ECM deposition, which will stimulate the activation of fibroblasts and further induce peritoneal fibrosis (R. Wang et al., 2022). Oxidative stress is a series of pathophysiological processes caused by the excessive production of ROS and other oxides in cells, which involves the generation, damage, repair, and other aspects of intracellular molecules. AGEs produced by peritoneal permeation can induce a series of chain reactions after binding with RAGE, which promotes the production of mitochondria and endoplasmic reticulum ROS, causing damage to peritoneal mesothelial cells. On the contrary, ROS can also increase the production of AGEs *in vivo*, and the cycle repeats and becomes vicious. In order to maintain ROS at a low level, the abundant antioxidant system in the body plays an important role, among which nicotinamide-adenine dinucleotide phosphate (NADPH) plays a regulatory role in ROS(Li and Shah, 2003; Lennicke and Cochemé, 2021). Most ROS also comes from mitochondria, which participate in cell metabolism through oxidative phosphorylation (Wang et al., 2011).

EMT is a dynamic structure composed of interstitial matrix and basement membrane, and its composition and proportion will change with different physiological and pathological states (Sutherland et al., 2023; Yuan et al., 2023), that is, when EMT occurs, epithelial cells lose cell polarity, intercellular adhesion, and the support of basement membrane, and at the same time acquire the characteristics of mesenchymal cells (Masola et al., 2022). In the process of fibrosis, transforming growth factor-β (TGF-β) is its main effector, which plays an important role in the occurrence and development of many diseases (such as liver fibrosis (Song et al., 2023), kidney lesions (Tang et al., 2022), pulmonary fibrosis (N. Zhang et al., 2023), etc.). TGF-β is a strong fibrogenic factor (Bottinger and Bitzer, 2002) and plays an important role in the production of fibrosis.

## 3 Chemical composition of Astragalus memeranaceus

**FIGURE 1:**
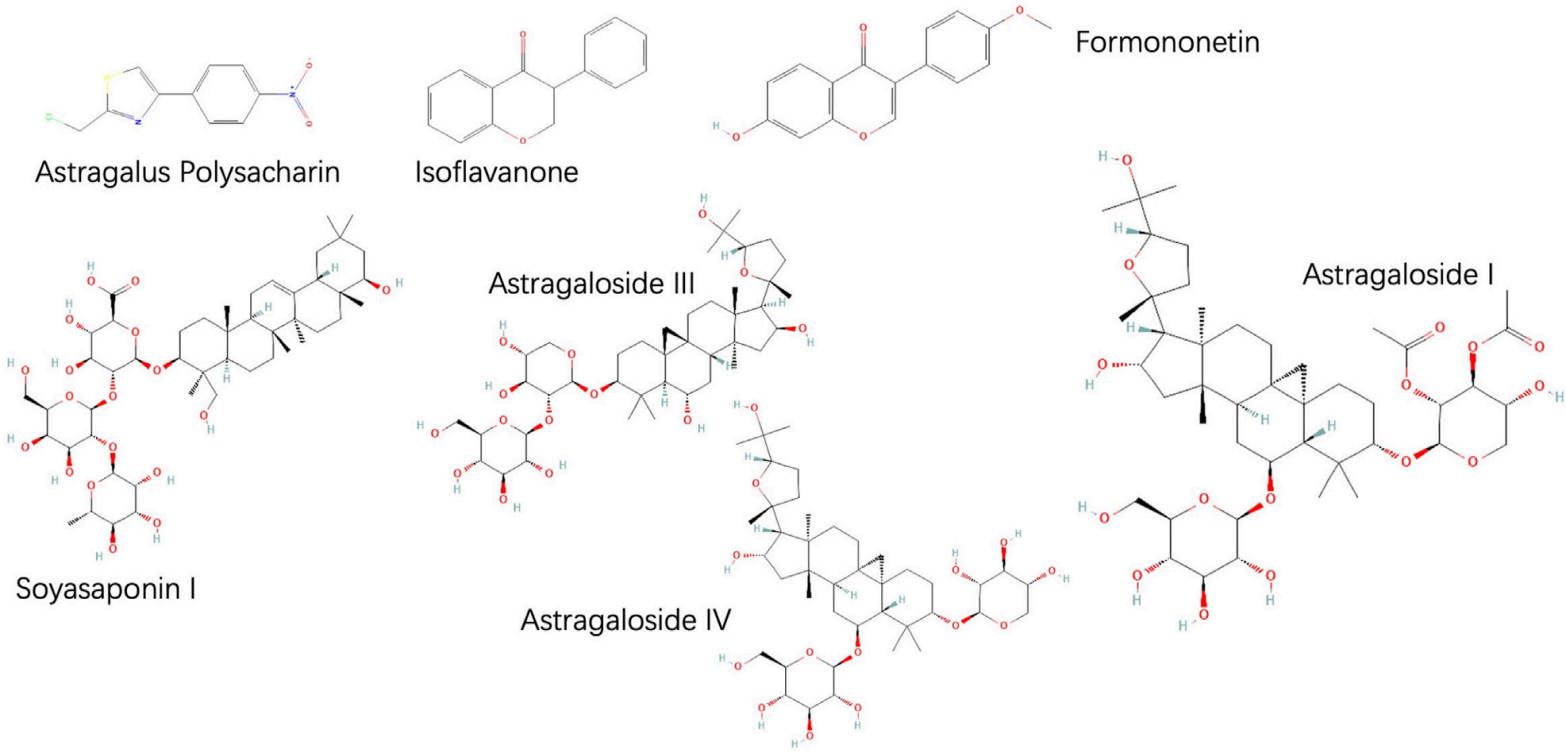
Chemical structures of a few bioactive constituents of Astragalus memeranaceus.

AMS, the root of legumes, was first recorded in Shennong’s Herbal Classic. AMS is the dried root of the perennial herbs *Astragalus membranaceus* (Fisch.) Bge. At present, Mongolian Astragalus is widely used. Its chemical composition is complex but consistent (T. Chen et al., 2021), (S. Li et al., 2020), containing polysaccharides, glycosides, flavonoids, proteins, amino acids, alkaloids, trace elements, and other active substances (Sheng et al., 2018; Sheik et al., 2021). At present, the extraction methods of saponins include traditional decoctions, reflux extraction, ultrasonic or microwave assisted extraction, bionic extraction, enzyme assisted extraction and so on (Sun et al. , 2023), 160 saponins have been found, triterpenoid saponins are the most studied secondary metabolites in this group of plants, of which Astragaloside IV (AS-IV) is the most common (Su et al. , 2021). At the same time, the results showed that the content of secondary metabolites of flavonoids varied greatly in different parts of the plant, and the root contained the most active components (Zhang et al., 2012; Sun et al., 2023), more than 60 kinds of flavonoids have been identified from AMS, including isoflavones, onononin, and vermus isoflavones (Liu et al. , 2017). Among them, saponins, flavonoids and Astragalus polysaccharide (APS) are the main active components. APS, as one of the main active components in AMS, has anti-inflammatory and immunomodulatory effects, which are important for the regulation of multiple systems and the treatment of multiple diseases (Cai et al., 2023). Due to the complex chemical structure of APS, its components are difficult to separate and identify (Shan et al. , 2019), So it was not studied in depth (Figure 1).

## 4 Effects of Astragalus memeranaceus and its active components on peritoneal fibrosis

It has been found that AMS and its active ingredients can improve the clinical manifestations of peritoneal permeation-induced PF. The biological activities of Astragalus and its active ingredients are shown in Table 1.

**TABLE 1:** Biological activities of Astragalus memeranaceus and its active components.

Biological activities	Astragalus membranaceus/Active ingredients	Bioassay	Results	References
Antioxidant activity	Astragalus membranaceus injections	Chemiluminescent probes	Reduce reactive oxygen species production	Xu et al., 2010; Liu et al., 2014
	Astragalus membranaceus IV	Chemiluminescent probes	Reduce mitochondrial membrane lipid peroxidation caused by high glucose dialysis solution by reducing the production of reactive oxygen species	Li et al. (2019)
			Reduce the production of reactive oxygen species to inhibit the phosphorylation of Smad2/3	Shi et al. (2016)
		Western blot	Protect cells by inhibiting iron death, the level of Fe^2+^ and reactive oxygen species decreased	Cao et al. (2021)
Immunomodulation	Astragalus membranaceus injections	Nitrate reductase assay	Antagonize the inhibition of abdominal dialysis on macrophage activity and phagocytosis function, and increase the secretion of tumor necrosis factor-α and nitrogen oxide	Zhang et al. (2007)
		TGF-β activation	Treatment with 10 mg/mL, reverse the overexpression of transforming growth factor-β1	Xu et al. (2005)
		Histology	Inhibit the action of monocyte chemotactic protein-1, and reduce the recruitment and activation of monocytes/macrophages	Li et al. (2014)
	Astragalus membranaceus	Wnt/β-catenin activation	Inhibition of β-catenin protein expression and inhibition of Snail transcription factor	Yu et al. (2018)
	Astragalus membranaceus IV	TGF-β activation	Upregulate Smad7, reverse the overexpression of transforming growth factor-β1	Li et al. (2013)
Anti-inflammatory activity	Astragalus membranaceus IV	Western blot	Partially restore the homeostasis of the low level of NLRP3 receptor protein 3 inflammasome activation	Zhang et al. (2015)
Anti-apoptosis	Astragalus total saponins	Western blot	Promote the expression of proliferator-activated receptor coactivator-1α and increase mitochondrial synthesis	Li et al. (2022)
	Astragalus polysaccharide	Western blot	Downregulate the protein expression of Bcl-2-associated X protein and cleaved Caspase3 and upregulate Bcl-2	Zhao, Shan, et al. (2020)

### 4.1 Impact of Astragalus memeranaceus on peritoneal fibrosis

The improvement effect of AMS on PF has been proved. Huang et al. (Huang et al., 2003) cultured HMrSV5 cells *in vitro* and found that a high concentration of AMS not only did not cause cytotoxicity, but also had a significant protective effect in reducing cell destruction, indicating that AMS could reverse the damage caused by high glucose abdominal permeate solution and inflammatory factors, and promote the proliferation of mesothelial cells, with an effect similar to that of steroid drugs. These results suggest that AMS is important for the maintenance of normal peritoneal function. One study randomly assigned 56 patients with continuous ambulatory peritoneal dialysis. By comparing abdominal dialysis before and after treatment, it was found that using abdominal dialysis containing AMS could antagonize the inhibition of abdominal dialysis on macrophage activity and phagocytosis function. At the same time, AMS can increase the secretion of TNF-α and nitrogen oxide, indicating the immune defense function of AMS in PF treatment (M. Zhang et al., 2007). In the process of TGF-β signaling, Smads protein family is a key mediator. When active TGF-β binds to the TGF-β receptor on the surface, it induces phosphorylation of the C-terminal of downstream Smad protein, phospho-Smad2 and Smad3 form heterodimeric complexes with Smad4, and transported to the nucleus (Roberts and Derynck, 2021). High glucose peritoneal dialysis solution can lead to excessive secretion of TGF-β1, and the use of a conventional dose of peritoneal dialysis solution containing AMS injection (10 mg/mL concentration) can reverse the overexpression of TGF-β1, suggesting that AMS plays a certain role in delaying PF through TGF-β (H. Xu et al., 2005). In this process, Smad7 acts as a TGF-β/Smad signaling antagonist to inhibit TGF-β-mediated phosphorylation of Smad2 and Smad3 (Silva et al., 2019). In the rat PF model induced by standard abdominal permeation solution, it was found that AMS injection (contained 2 g/mL crude drug) can alleviate PF, and the mechanism is to inhibit the action of monocyte chemotactic protein-1, thereby reducing the recruitment and activation of monocytes/macrophages. In addition, the expression of TNF-α and TGF-β1 in the peritoneum was reduced after AMS treatment at a dose of 4000 mg/kg, and the reduction of phosphorylated SMAD2/3 also confirmed the inhibitory effect of AMS on TGF-β/Smad pathway (Z. Li et al., 2014), and it was also found that the role of macrophages in peritoneal fibrosis is bidirectional and complex.

The activation marker of the Wnt/β-catenin signaling pathway is the upregulated expression and nuclear translocation of the key protein β-catenin, and Snail is its transcription factor (Schunk et al., 2021). EMT of HMrSV5 cells was induced by TGF-β, and β-catenin and Snail were reduced. After AMS intervention, EMT was further inhibited. The results suggested that AMS could inhibit TGF-β-induced EMT of HMrSV5 cells to a certain extent. Part of the mechanism is through activation of Wnt/β-catenin signaling pathway, inhibition of β-catenin protein expression and inhibition of Snail transcription factor. In addition, Glycogen Synthase Kinase-3 (GSK-3β) can exist in complex form with β-catenin. Some scholars further detected the expression and phosphorylation of GSK-3β and β-catenin proteins in the cytoplasm and nucleus of peritoneal mesodermal cells after AMS intervention, and found that AMS could stabilize GSK-3β and β-catenin complex and degrade β-catenin. The specific mechanism of AMS regulating Wnt/β-catenin signaling pathway was reconfirmed and further discovered (Yu et al., 2018). Wnt/β-catenin signaling pathway can crosstalk with TGF-β signaling pathway (Guo et al., 2017). Based on this, further experiments found that β-catenin can bind to Smad7 protein. It was found that β-catenin may compete with Smad7 after the construction of Smad7 cell lines, and at the same time reduce the inhibitory effect of AMS on EMT to a certain extent, and the same operation was performed in the rat PF model induced by intrapitoneal injection of 4.25% high glucose peritoneal permeate solution, and the results were also similar. It is suggested that AMS can upregulate Smad7 and competitively inhibit β-catenin through both classical Wnt/β-catenin pathway and crosspoilers with TGF-β, and finally reduce β-catenin expression and interfere with PF(Yu et al., 2018). Some studies have found that stimulation of rat peritoneal mesothelial cells with AMS medium can antagonize the upregulation of ROS and NADPH oxidase subunit p47phox expression caused by Angiotensin Ⅱ, and not only reduce ROS production and p47phox expression. It can also inhibit the expression and activity of NADPH oxidase, so it can be inferred that AMS can reduce the production of ROS by inhibiting the activity of NADPH oxidase, thus blocking its signal transduction, inhibiting the overexpression of TGF-β1 downstream, reducing the occurrence of EMT, and delaying PF (J. Xu et al., 2010). In addition, the experiments of Liu et al. (X. Liu et al., 2014) showed that TGF-β significantly induced the expression of NADPH oxidase subunit p67phoxmRNA and protein in rat peritoneal mesothelial cells, and induced intracellular ROS production, which could be significantly improved by AMS stimulation.

**FIGURE 2:**
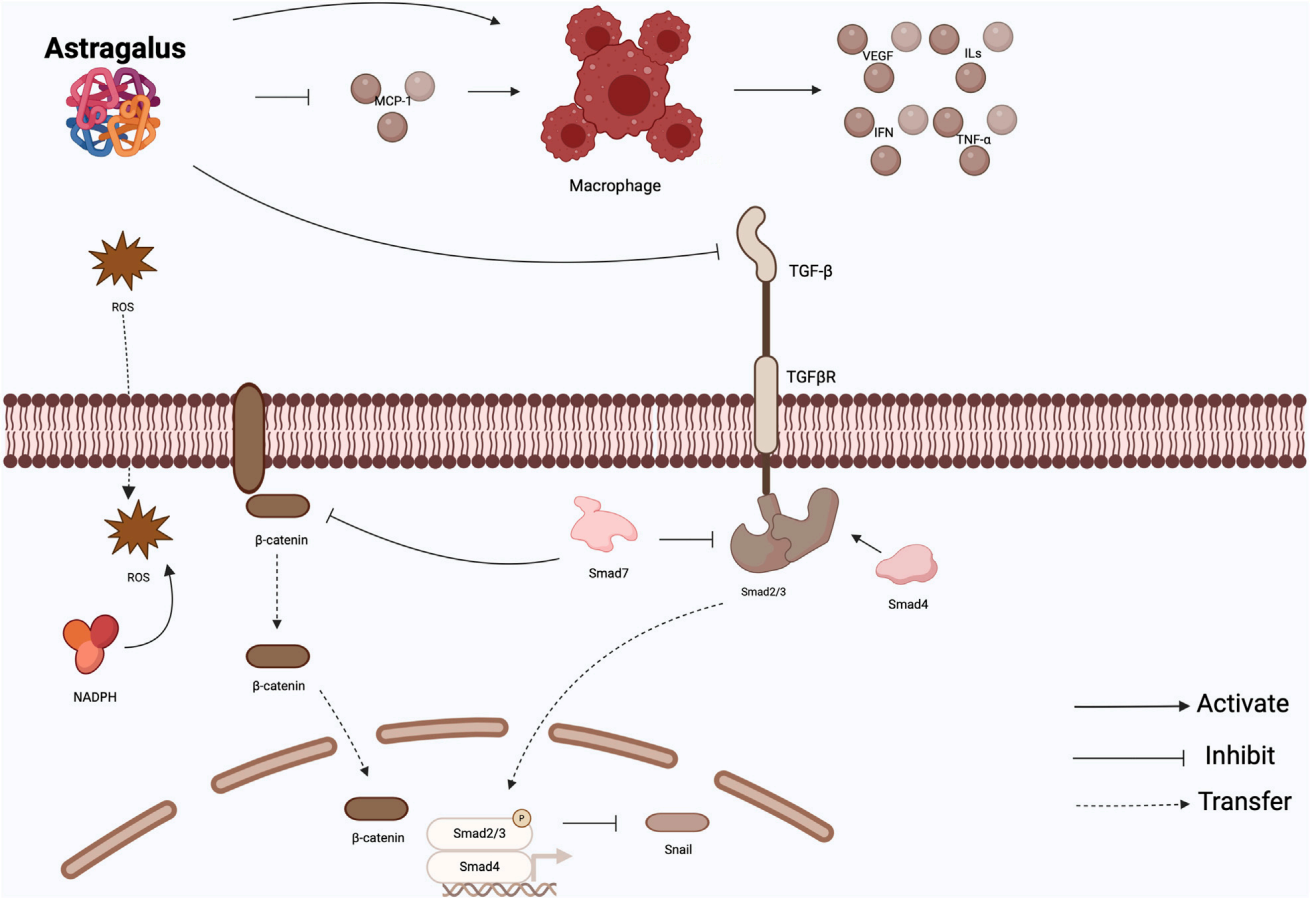
Effect of Astragalus memeranaceus. AMS not only antagonizes the effect of peritoneal dialysate by restoring macrophage activity, but also inhibits MCP-1 to reduce macrophage recruitment and activation. At the same time, it can inhibit TGF-β pathway and Wnt/β-catenin-related pathway. It also reduces the production of ROS. MCP-1: monocyte chemotactic protein-1; TGF-β: transforming growth factor-β; ROS: reactive oxygen species.

In conclusion, a certain concentration of AMS not only does not cause damage to the body, but also has a protective effect on PF, which is specifically reflected in the fact that AMS can act on the target of TGF-β, interfere with EMT through the associated cytokine network, and improve the oxidative stress in the body to balance the production of ROS, Further, regulate the downstream TGF-β related signaling pathway. However, the research direction of AMS is too simple and not deep enough, only stays in the upstream medium. At the same time, for other cytokines, such as connective tissue growth factor and other downstream mediators, these studies are not clear. Therefore, the mechanism of AMS regulating PF still needs to be further studied (Figure 2).

### 4.2 Influence of Astragalus saponin on peritoneal fibrosis

AS-IV, isoastragalus Ⅰ, acetoflavin Ⅰ, Fusarium flavin A, Fusarium flavin B and soybean saponin Ⅰ were isolated from Astragalus memeranaceus. In TGF-β-induced EMT models, AS-IV decreased the expression of vimentin and phospho-Smad2/3 in a dose-dependent manner and increased the expression of Smad7. Therefore, it is speculated that AS-IV can alleviate PF by up-regulating Smad7 in TGF-β/Smad signaling pathway (Z. Li et al., 2013). In addition to the classical Smad pathway, TGF-β can also promote cell survival, proliferation, and collagen synthesis by activating the PI3K/Akt pathway (Y. E. Zhang, 2009). Yu et al. found that TGF-β1 upregulates β-catenin when inducing EMT in HMrSV5 cells, while AS-IV can degrade β-catenin to a certain extent. β-catenin is not only involved in stabilizing cell adhesion, but also a key signaling molecule in the Wnt signaling pathway. At the same time, AS-IV can inhibit the high expression of p-Akt, p-GSK-3β and p-mTOR. Compared with Akt inhibitor MK226, mTOR inhibitor rapamycin and Akt pathway agonist Insulin, it is found that AS-IV is likely to delay PF through the AKt pathway (Yu et al., 2019).

NLRP3 receptor protein 3 inflammasome (NLRP3 inflammasome) is a multiprotein complex, located in the cytoplasm and is named by NOD-like receptor protein 3 (NOD-like receptorprotein 3, NLRP3 inflammasome). NLRP3, apoptosis-associated speck-like potein containing a CARD, and cysteinyl aspartate specific proteinase-1 (Caspase-1), and the activation of NLRP3 inflammasome on the one hand will lead to the release of pro-inflammatory mediators such as interleukin1-β (IL-1β) and interleukin-18, this can induce the progression of inflammation (Lamouille et al., 2014), on the other hand, also can induce the scorch death of damaged cells (J. Chen and Chen, 2018). Zhang et al. (L. Zhang et al., 2015) found that high-glucose peritoneal dialysis solution could activate NLRP3 inflammasome-related parts in a time-dose-dependent manner, such as NLRP3, pro-caspase-1 precursor containing cysteine, IL-1β precursor and IL-1β. AS-IV can partially inhibit peritoneal mesothelial-to-mesenchymal transition by blocking its activation in a concentration-dependent manner. At the same time, the inhibition of the NLRP3 inflamome by AS-IV is not the same as the complete block of other small molecule compounds (such as dopamine receptor-1), but is more likely to partially restore the homeostasis of the low level of NLRP3 activation.

By establishing an *in vitro* HPMCs/HMrSV5 cell model, Li et al. (Z. Li et al., 2019) confirmed that AS-IV can reduce mitochondrial membrane lipid peroxidation caused by high glucose dialysis solution by reducing the production of ROS, thus alleviating the disorder of charge distribution inside and outside mitochondria, protecting mitochondrial function, improving oxidative stress, and ultimately inhibiting apoptosis induced by mitochondrial damage. On the other hand, AS-IV can control the expression of the translocator of the outer mitochondrial membrane (Tom), although it has no significant effect on the expression of Tom20. However, Tom70 can be upregulated to reverse the damage caused by high glucose dialysate and protect the mitochondrial translocase system. It has also been observed that AS-IV could reduce the expression levels of dynamin related protein-1 and recombinant human mitochondrial fission protein. It also promoted the expression of optic atrophy rotein, a mitochondrial fusion protein. Therefore, it is speculated that AS-IV can improve HPMCs injury and protect peritoneal structure and function through the above pathways. In addition, experiments have shown that ROS may act AS a second messenger to regulate the Smads signaling pathway and mediate the occurrence of EMT, and AS-IV can reduce the production of ROS to inhibit the phosphorylation of Smad2/3, thus achieving the purpose of inhibiting and reverting EMT, similar to the ROS scavenger n-acetylcysteine (NAC) (Shi et al., 2016).

Ferroptosis is a cell death mode different from apoptosis, necrosis, and autophagy, and its main feature is induced lipid peroxidation (Stockwell et al., 2017). Cao Huimin et al. (Cao et al., 2021) found that there was iron death in the HMrSV5 model, and AS-IV could protect cells by inhibiting iron death. In this study, it was found that after the intervention of AS-IV, the level of Fe^2+^ and ROS decreased, the content of MDA decreased, and the content of GSH increased. The expression of cell tumor antigen p53 and Acyl-CoA synthetase long-chain family member 4 was downregulated. The expression of solute carrier family 7 member 11 (SLC7A11,xCT)and glutathione peroxidase 4 (GPX4) was upregulated. It is suggested that AS-IV may protect peritoneal cells by reducing Fe2+ content, inhibiting ROS production and enhancing xCT and GPX4 activity through cystine/glutamate reverse transporter (xCT)/GPX4 pathway.

**FIGURE 3:**
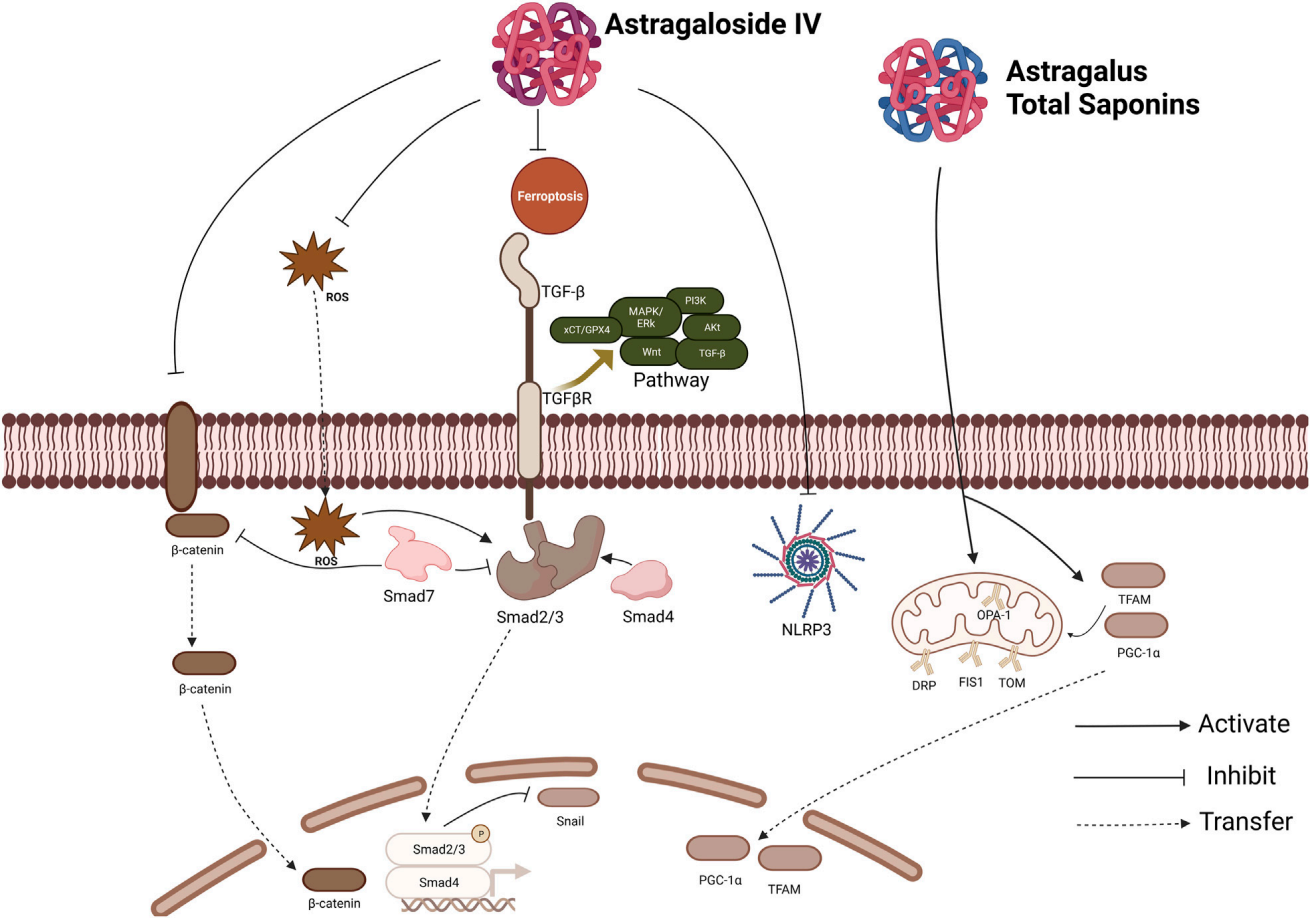
Effect of Astragaloside IV and Astragalus total saponins. The inhibition of AS-IV involves several pathways, including TGF-β pathway, PI3K pathway, Akt pathway, MAPK pathway, etc. It can inhibit Ferroptosis and the production of ROS and inhibit the NLRP3 Inflammasome. TGF-β: Transforming Growth Factor-β; ROS: reactive oxygen species; NLRP3: NOD-like receptor protein 3.

Proliferator-activated receptor coactivator-1α (PGC-1α) is an important transcription coactivator of cell kernel receptors. Its primary function is to regulate the synthesis of mitochondrial proteins, including subunits of the respiratory chain complex, by activating nucleus respiratory factor 1 (NRF-1). In addition, PGC-1α binds to transcription factor mitochondrial A, TFAM, thereby initiating the replication and transcription of mitochondrial DNA (Feng et al., 2016). Some studies (Z.-H. Li et al., 2022) suggested that the expression of Bcl-2 in rat stromal cells with fibrosis induced by high glucose was inhibited, and similar changes were also observed in the expression of PGC-1α, NRF-1 and TFAM. However, after Astragalus total saponins (ATS) treatment, the expression of these proteins increased, and after interfering with PGC-1α treatment, the expression of ATS on cell viability and mitochondrial number, including related proteins, showed a downward trend. Therefore, it is speculated that the intervention mechanism of ATS is to promote the expression of PGC-1α and increase mitochondrial synthesis, thereby inhibiting apoptosis and fibrosis-related proteins. Reduce fiber collagen synthesis and fiber tissue proliferation, thereby reducing the degree of PF (Figure 3).

In conclusion, Astragalus saponins (including AS-IV), AS-IV, the main active ingredient of AMS, can delay fibrosis by regulating oxidative stress, apoptosis and inhibiting inflammatory response.

### 4.3 Impact of Astragalus polysaccharide on peritoneal fibrosis

It has been shown that high glucose peritoneal permeate decreases the mitochondrial membrane potential of HMrSV5, and APS can reverse this process. In addition, APS can also downregulate the protein expression of Bcl-2-associated X protein and cleaved Caspase3 and upregulate Bcl-2, antagonizing cell apoptosis and protecting peritoneal tissue, and it is confirmed that APS is associated with the expression level of proteins related to mitochondrial apoptosis of HMrSV5 (Zhao, Shan, et al., 2020). Vascular endothelial growth factor (VEGF) can induce endothelial cell proliferation and promote angiogenesis. Appropriate angiogenesis can promote tissue repair, but when the degree of angiogenesis exceeds a certain limit, the peritoneal glucose absorption rate will increase, resulting in the disappearance of peritoneal osmotic concentration gradient and the reduction of peritoneal ultrafiltration. On this basis, some scholars have found that APS can inhibit the expression of VEGF protein level, thereby inhibiting angiogenesis and reversing the occurrence of fibroperitoneal maintenance in PD rats (Feng et al. , 2024) In conclusion, APS protects peritoneal tissue mainly by regulating mitochondria-related proteins and VEGF. Recently, it has been reported in the literature that APS can inhibit the TLR4/NF-κB signalling pathway and reduce apoptosis, thus ameliorating the occurrence of idiopathic pulmonary fibrosis in mice. Surprisingly, the authors also found that the gut microbiota can also affect lung disease through the gut-lung axis, and APS can increase the proportion of intestinal probiotics, reduce harmful bacteria, and balance the gut microbiota by regulating the metabolic pathway, which ultimately leads to therapeutic effects. This exists as an inspiration for us to study the treatment of PF (Wei et al., 2023).

## 5 Summary and outlook

AMS belongs to traditional Chinese medicine. Existing literature has shown that AMS and its active components have certain efficacy in delaying PD-induced PF, and its potential mechanism is related to anti-inflammatory response, inhibition of oxidative stress, regulation of apoptosis, etc., involving a variety of signaling pathways, such as TGF-β pathway, Wnt pathway and AKT pathway. In this paper, the role of AMS and its active components in anti-peritoneal dialysis-related peritoneal fibrosis was reviewed in order to provide new drug targets. However, the available research is not comprehensive. Although the toxicity of traditional Chinese medicine is relatively low, the active ingredients of AMS are complex, the bioavailability and conversion rate are low, the pharmacokinetic mechanism is unknown, and its preparations are difficult to promote in clinical practice (Cai et al., 2023). Fortunately, there have been no reports of toxic effects of AMS and its active ingredients. AMS was well tolerated in most patients. Therefore, in a certain dose range, the preparation of concentrated pills may become a method for clinical application. At present, there are many researches on the pharmacology of AMS and its active ingredients, as well as its compound Chinese medicine preparations. Although the researches on the mechanism of action of AMS extract or single physiological active ingredient are extensive, but not in-depth. Perhaps, it is also a method to use AMS in combination with the original drug, or to deliver it to the lesion site through a drug delivery carrier such as hydrogel through loaded monomer to achieve the purpose. At the same time, the research on PD-related PF is not deep enough, and only stays at the level of protein expression, and there are few studies on gene expression. Therefore, the potential targets and binding sites of AMS for PF treatment can be identified through pharmacological research methods and molecular docking techniques, and verified with gene knockout or overexpression techniques, or small molecule inhibitors or agonists.

In summary, AMS and its active components can inhibit the occurrence and development of PD-related PF. In the future, combined with CM theory, serum pharmacology and nanoimaging technology, the pharmacological mechanism of AMS in the treatment of PF will be comprehensively studied.
